# Supplementary material for: Saccharomyces boulardii (CNCM I-745) improves intestinal damage in sepsis by remodeling intestinal flora structure
Source: Front Cell Infect Microbiol. 2025 Jul 31;15:1602792. doi: 10.3389/fcimb.2025.1602792 (PMC12350329; doi:10.3389/fcimb.2025.1602792)
Supplement: Supplementary file 1 [file DataSheet1.zip › summary/summary/6_Taxonomy_community/1_Taxonomy_profiling/1_Domain/All/Domain_abund_top30.sample.cluster.pdf]

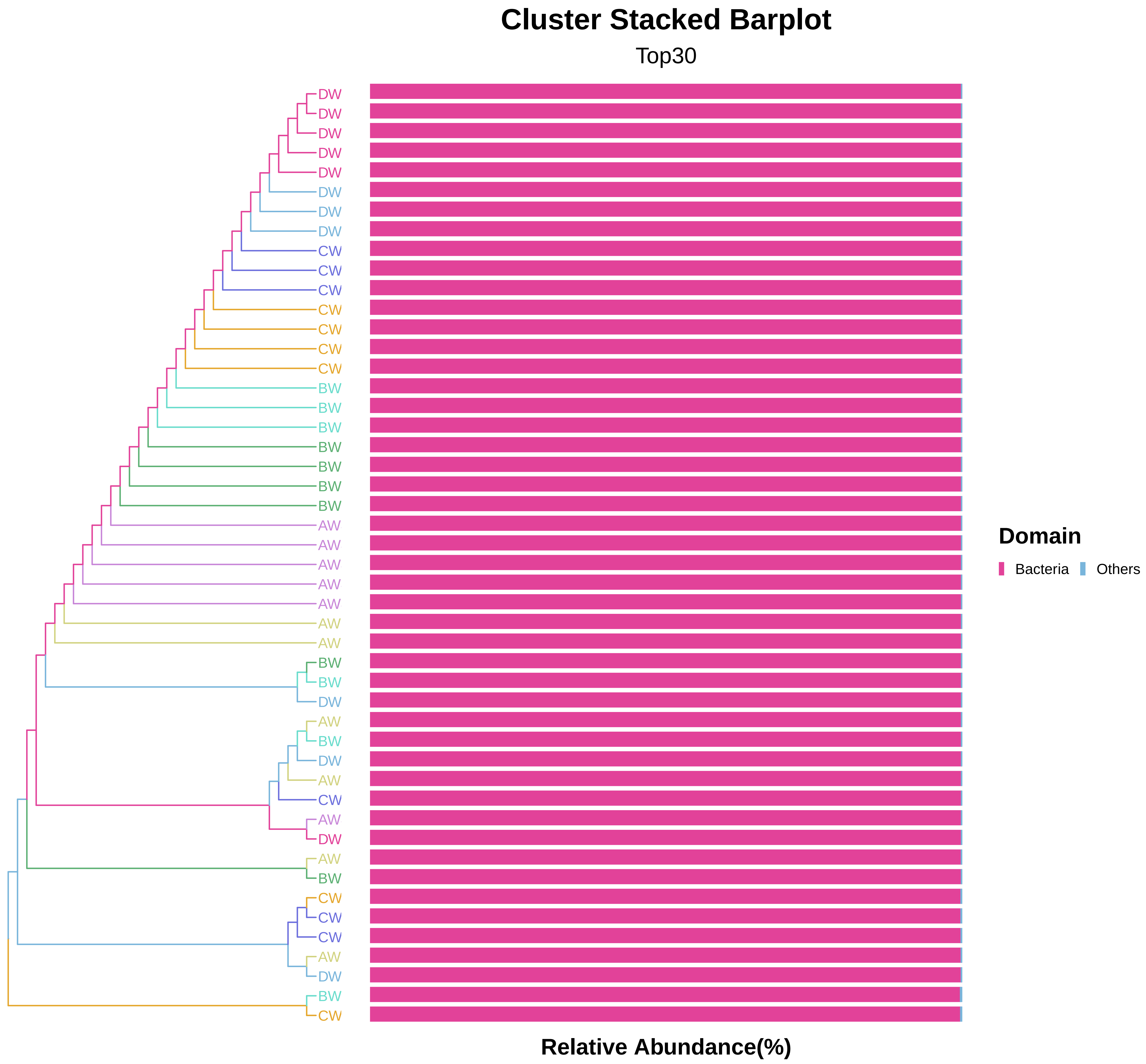
Cluster Stacked Barplot
Top30
DW
DW
DW
DW
DW
DW
DW
DW
CW
CW
CW
CW
CW
CW
CW
BW
BW
BW
BW
BW
BW
BW
AW
AW
AW
AW
AW
AW
AW
BW
BW
DW
AW
BW
DW
AW
CW
AW
DW
AW
BW
CW
CW
CW
AW
DW
BW
CW
Relative Abundance(%)
Domain
Bacteria
Others
